# Barriers to Addressing Sexual Health Needs of Adults With Chronic Kidney Disease: A Multidisciplinary Survey of Renal Healthcare Professionals

**DOI:** 10.1111/jorc.70087

**Published:** 2026-11-10

**Authors:** Clare McKeaveney, Xingge Sun, James McMahon, Ann Bonner, Maoliosa Donald, Sandra Dumanski, Meghan Elliott, Jeanette Finderup

**Affiliations:** ^1^ School of Nursing and Midwifery, Queen's University Belfast Belfast UK; ^2^ School of Nursing and Midwifery, Griffith University Brisbane Australia; ^3^ Kidney Health Service, Metro North Health Brisbane Australia; ^4^ Department of Community Health Sciences Cumming School of Medicine, University of Calgary Calgary Canada; ^5^ University of Calgary Calgary Canada; ^6^ Department of Medicine University of Calgary Calgary Canada; ^7^ Department of Community Health Sciences University of Calgary Calgary Canada; ^8^ Department of Renal Medicine Aarhus University Hospital Aarhus Denmark; ^9^ Department of Clinical Medicine Aarhus University Aarhus Denmark

**Keywords:** healthcare professionals, kidney disease, quantitative study, sexual health

## Abstract

**Background:**

Sexual dysfunction is common among adults with chronic kidney disease and can significantly affect health and well‐being. Understanding healthcare professionals' attitudes, training experiences, and perceived barriers toward discussing sexual health needs with patients is essential to improving patient care.

**Methods:**

A cross‐sectional survey was conducted with healthcare professionals involved in the care of adults with chronic kidney disease. The survey comprised four sections aimed to uncover participants' attitudes and beliefs toward sexual health, experience of sexual health training and practice, and barriers to discussing sexual health. Recruitment and data collection were conducted through social media and professional renal networks between December 2022 and April 2023. Descriptive statistics (means, standard deviations, frequencies, and percentages) were employed to summarise responses. Bivariate analyses were conducted to examine differences in sexual health attitude and belief scores across participants' age, sex, and professional experience. Open‐ended responses were summarised descriptively.

**Results:**

A total of 135 participants completed the survey. Most were female nurses aged 35–54, working in haemodialysis, with more than 10 years' experience. Almost 80% had not received any sexual health training in their current or previous roles. The average attitude score was 35.49 ± 6.95, suggesting a relatively positive attitude. Ninety percent believed that addressing sexual health is essential for optimising patients' health outcomes. Key barriers included a lack of training, insufficient knowledge, and cultural or religious considerations.

**Conclusion:**

Training for healthcare professionals on addressing sexual health in renal care is limited. Educational initiatives are required to improve professionals' confidence and competence in discussing sexual health.

## 1 Introduction

Sexual dysfunction is an abnormality in arousal, libido, sexual activity, orgasm, or sexual satisfaction (Agarwal and Pavlakis 2021), and is prevalent in all stages of chronic kidney disease (CKD), including after kidney transplantation (van Ek et al. 2017a, 2017b; Mota et al. 2019; Harrison et al. 2020). Men and women with kidney failure receiving kidney replacement therapy are likely to have a higher prevalence of sexual dysfunction than their healthy peers (Luo et al. 2020). While kidney transplantation could alleviate some aspects of sexual dysfunction due to the return of normal kidney function (Pruett et al. 2024), the use of immunosuppressive medications, such as sirolimus and steroids, and the fact that steroids can change body image, adults are likely to experience an impact on sexual function and sexual satisfaction (Agarwal and Pavlakis 2021). The most reported sexual dysfunctions are erectile dysfunction in men and decreased vaginal lubrication, pain during intercourse, and difficulty reaching orgasm in women (Abarca‐Durán et al. 2021; Pyrgidis et al. 2021a, 2021b).

Sexual dysfunction can have a significant impact on the holistic health of individuals, and can cause psychosocial complications such as depression, anxiety, poor self‐esteem, low confidence in relationships, and poor health‐related quality of life (Mota et al. 2019; Cruz et al. 2022; Ali and Dave 2020). However, sexual health issues are not routinely assessed and addressed by renal nurses and clinicians due to confusion about roles and responsibilities in sexual health matters, limited education and training, a shortage of private space for discussion, and personal barriers such as embarrassment (Hendren et al. 2019; McGrath et al. 2021; Mckie et al. 2024).

### 1.1 Literature Review

In the last 10 years, there has been a lack of research reporting on the perspectives of multidisciplinary renal healthcare teams discussing sexual health issues with patients (van Ek et al. 2015, 2017a, 2017b; Yodchai et al. 2018; Mckie et al. 2021). The most recent survey conducted (Mckie et al. 2024) included 65 Australian nurses, exploring their perspectives of discussing sexual health in adults receiving haemodialysis. Notably, the responsibilities of providing sexual health care for adults with CKD ought to be multidisciplinary, involving not only nurses but also other healthcare professionals (HCPs). Previous research has argued that there is no detailed multidisciplinary protocol that underpins sexual health care for patients with CKD in most nephrology departments (van Ek et al. 2015). Thus, it is imperative to uncover the current sexual health provision for adults with kidney disease from HCPs, understand HCP competence in delivering such care, and identify potential barriers that may hinder effective practice. This study aimed to investigate the attitudes and barriers faced by renal HCPs in providing sexual health assessment and support to adults with CKD. This aim was achieved through three objectives to:

- Understand the exposure to sexual health education or training for renal HCPs.
- Evaluate renal HCPs attitudes and beliefs toward assessing the sexual health of adults with CKD.
- Assess the perceived barriers to discussing sexual health with adults with CKD.

## 2 Method

### 2.1 Study Design

A cross‐sectional survey design was employed. This study was reported in accordance with the Strengthening the Reporting of Observational Studies in Epidemiology guidelines (von Elm et al. 2007).

### 2.2 Participants

Eligible participants were HCPs, 18 years of age or older, from any health discipline and country, who were responsible for providing healthcare to adults with CKD of any stage. Students and unqualified staff were not eligible to participate.

### 2.3 Survey Instrument

The survey comprised four sections. The first section gathered sociodemographic characteristics. Section two contained the Sexual Attitudes and Beliefs Survey (SABS). This 12‐item survey aims to assess attitudes and beliefs of HCPs towards addressing patients' sexuality and sexual health (Reynolds and Magnan 2005). Participants were required to respond to each question on a 6‐point Likert scale from strongly disagree (1) to strongly agree (6). Total scores range from 12 to 72, with higher scores presenting more negative sexual attitudes and beliefs. Cronbach alpha testing of the instrument (Azar et al. 2022) has demonstrated an acceptable to good internal consistency (Cronbach *α* = 0.75 and 0.82). SABS scores are typically interpreted on a continuum. In the absence of formal categorisation, a theoretical midpoint of the scale (42) was set as a neutral reference point. Participants with scores below and above this reference point were viewed as possessing either positive or negative attitudes and beliefs towards sexuality and sexual health, respectively.

Section three aimed to uncover HCPs' experiences of sexual health training and practices. Developed by the research team, and using binary answers (yes/no), HCPs were questioned as to whether they have ever participated in a sexual health education or training programme in either their current or previous clinical role. In addition, the frequency of collecting patients' sexual histories was uncovered using a 5‐point Likert scale from never (1) to always (5). The willingness to attend sexual health training and alternative HCPs for discussing sexual health issues was also surveyed. The final section aimed to uncover the perceived barriers to discussing patient sexual concerns. This section comprised 15 items identified through the literature (Reynolds and Magnan 2005, quoted in Azar et al. 2022). Utilising a 6‐point Likert scale, each item is graded from strongly disagree (1) to strongly agree (6). A mean agreement score was calculated for each item, with a higher score reflecting a greater perceived barrier to assessing patients' sexual health. This section ended with an open‐text question to provide participants with an opportunity to highlight any additional barriers not listed in the preceding questions.

### 2.4 Recruitment and Study Procedures

The survey was circulated through social media (e.g., Facebook) and via professional renal networks (e.g., the European Dialysis and Transplant Nurses Association/European Renal Care Association (EDTNA/ERCA)). Access to the survey, attached to a weblink, was granted only once the participant had read the information sheet and provided consent to participate. Survey data was collected between December 2022 and April 2023. The study used non‐probability convenience sampling to recruit participants. As the total global population of HCPs involved in caring for those with CKD is unknown, the target recruitment sample was 385 based on a large population value using the Raosoft calculator, assuming a 50% prevalence, 5% margin of error, and 95% confidence level.

### 2.5 Data Analysis

Data was analysed using the Statistical Package for Social Sciences 31. Data from incomplete surveys were removed. Descriptive statistics were used to summarise responses to each question, involving means, standard deviations (SD), frequencies, and percentages. In addition, mean participant SABS scores underwent bivariate analysis through the independent‐samples *t*‐test, and one‐way ANOVA to examine the differences in SABS scores across participant characteristics, including age, sex, education level, country, marital status, sex orientation, occupation and years of professional experience. Post hoc testing was applied where a statistically significant ANOVA result was found. Open‐ended responses were reviewed descriptively to provide additional context and identify commonly reported issues. No formal qualitative coding or thematic analysis was undertaken; therefore, these responses were used to illustrate findings rather than generate qualitative themes.

## 3 Results

### 3.1 Participant Demographics

A total of 135 participants were included in the analysis. Most respondents were aged between 35 and 54 (*n* = 79, 59%), female (*n* = 113, 84%), White/Caucasian (*n* = 118, 87%), married (*n* = 86, 64%), heterosexual (*n* = 121, 90%), with a postgraduate level education (*n* = 94, 70%). A detailed overview of participant characteristics can be found below (Table 1). The participants were recruited from 20 countries. Most respondents were from Denmark (*n* = 62, 46%) followed by the United Kingdom (*n* = 24, 18%) and Spain (*n* = 7, 5%) (Figure 1). Health disciplines included medicine, nursing, social work, dietetics and psychology, working in pre‐dialysis clinics, haemodialysis units, peritoneal dialysis (home therapies), conservative management, or post‐transplant clinics. Most participants were nurses (*n* = 114, 84%), working in haemodialysis units (*n* = 86, 64%), with over 10 years' experience in renal healthcare (*n* = 91, 68%).

**Table 1:** Participant characteristics.

Characteristic	Frequency (%)
Age	
18–24	6 (4%)
25–34	22 (16%)
35–44	32 (24%)
45–54	47 (35%)
55–64	25 (19%)
65 and over	3 (2%)
Sex	
Female	113 (84%)
Male	22 (16%)
Ethnicity	
White/Caucasian	118 (87%)
Asian/Pacific Islander	10 (7%)
Black or African American	1 (0.7%)
Hispanic	1 (0.7%)
Multiple ethnicity	3 (2%)
Other	2 (2%)
Marital status	
Married	86 (64%)
Single	26 (19%)
Divorced	9 (7%)
Separated	3 (2%)
Common Law Partnership	11 (8%)
Sexual orientation	
Straight or heterosexual	121 (90%)
Gay or Lesbian	4 (3%)
Bisexual	7 (5%)
Prefer not to say	3 (2%)
Education level	
Undergraduate or equivalent	41 (30%)
Postgraduate	94 (70%)
Occupation	
Nurse	114 (84%)
Nephrologist	9 (7%)
Social worker	1 (0.7%)
Dietician	3 (2%)
Psychologist	1 (0.7%)
Other	7 (5%)
Specialised department	
Pre‐dialysis	9 (7%)
Haemodialysis	86 (64%)
Peritoneal dialysis (home therapies)	9 (7%)
Conservative management	2 (2%)
Post‐transplant	8 (6%)
Other	21 (16%)
Years' experience in renal	
Less than 1 year	9 (7%)
1–5 years	21 (16%)
6–10 years	12 (9%)
More than 10 years	91 (68%)

**Figure 1:**
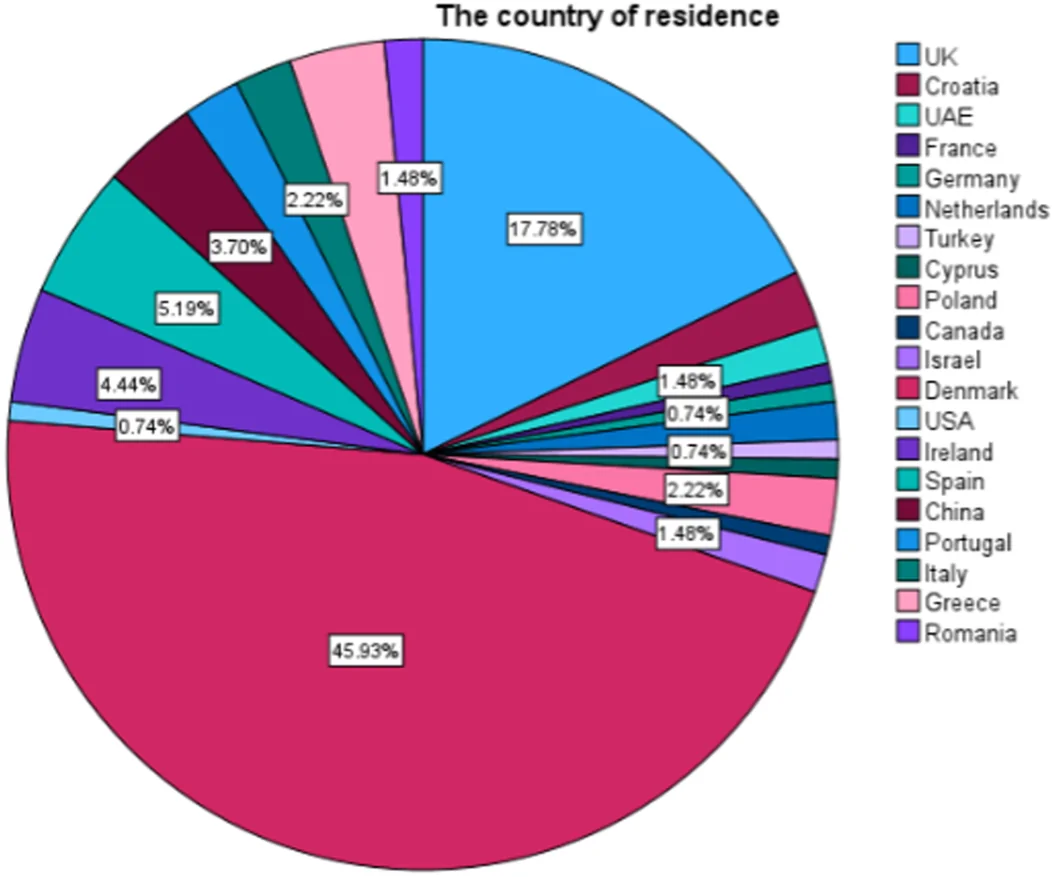
The country of residence for survey participants.

### 3.2 Sexual Health Training

Approximately 80% (*n* = 105) of HCPs had no experience with sexual health training in either their previous or current roles. Further, 63% of HCPs reported that they ‘rarely’ experienced sexual health history taking in clinical practice, in either their current (*n* = 43) or previous role (*n* = 42). The most common referrals by HCPs regarding sexually related questions were to a general practitioner, psychologist, nurse, or counsellor. Positively, 82% of HCPs (*n* = 111) would be willing to engage in sexual health training (Table 2).

**Table 2:** HCP experience of sexual health training and practices.

Experience of sexual health training and practices	Frequency (%)
*Sexual health training*	
In current role	
Yes	30 (22%)
In previous role	
Yes	30 (22%)
*Sexual health history taking*	
In current role	
Always	2 (2%)
Frequently	9 (7%)
Sometimes	31 (23%)
Rarely	43 (32%)
Never	50 (37%)
In previous role	
Always	2 (2%)
Frequently	8 (6%)
Sometimes	24 (18%)
Rarely	42 (31%)
Never	59 (44%)
*Willingness to participate in sexual health training*	
Yes	111 (82%)
Whenever patients ask me a sexually related question, I advise them to discuss the matter with an alternative HCP	
HCPs	General practitioners, Medical specialists, Psychologists, Nurses, Counsellors

### 3.3 Sexual Attitudes and Beliefs Survey

The average total SABS score of 35.49 ± 6.95 which was below the pre‐defined reference point, suggesting relatively more positive attitudes and beliefs among HCPs towards discussing sexual issues with adults living with CKD. The mean scores for each question range from 2.13 ± 1.14 to 3.86 ± 1.23 (Table 3). The statement most agreed upon was ‘Discussing sexuality is essential to patients' health outcomes' with 90.3% (*n* = 122) participant agreement, followed by ‘I understand how my patients' diseases and treatment might affect their sexuality’ with 89.7% (*n* = 121) agreement. The statements with the least agreement were ‘Sexuality is too private an issue to discuss with patients’ and ‘Whenever patients ask me a sexually related question,’ I advise them to discuss the matter with an alternative HCP, with 14.8% (*n* = 20) and 24.4% (*n* = 33), respectively.

**Table 3:** Sexual attitudes and beliefs survey.

Sexual Attitudes and Beliefs Survey (SABS)	Mean ± SD	Agree (%)	Disagree (%)
1. Discussing sexuality is essential to patients' health outcomes	2.27 ± 0.98	90.3	9.7
2. I understand how my patients' diseases and treatment might affect their sexuality	2.13 ± 1.14	89.7	10.3
3. I am uncomfortable about talking about sexual issues with patients	3.32 ± 1.36	51.8	48.2
4. I am more comfortable talking about sexual issues with my patients than are most HCPs I work with	3.37 ± 1.19	56.4	43.6
5. Most hospitalised patients are too sick to be interested in sexuality	3.00 ± 1.18	33.3	66.7
6. I make time to discuss sexual concerns with my patients	3.86 ± 1.23	39.2	60.8
7. Whenever patients ask me a sexually related question, I advise them to discuss the matter with an alternative HCP	2.68 ± 1.19	24.4	75.5
8. I am confident in my ability to address patients' sexual concerns	3.52 ± 1.23	54.8	45.2
9. Sexuality is too private an issue to discuss with patients	2.26 ± 1.22	14.8	85.2
10. Giving a patient permission to talk about sexual concerns is a HCP's responsibility	2.64 ± 1.32	77.1	22.9
11. Sexuality should be discussed only if initiated by the patient	2.72 ± 1.36	25.9	74.1
12. Patients expect HCPs to ask about their sexual concerns	3.71 ± 1.14	40.1	59.9
Total	35.49 ± 6.95		

Total SABS scores did not differ significantly according to sex, age, education level, country, marital status, sexual orientation, or occupation. Notably, there was a statistically significant difference in mean SABS scores according to years of professional experience and sexual health training. Post hoc tests indicated a significant difference in mean SABS scores as HCPs with over 10 years' experience had relatively more positive attitudes and beliefs than those with 1–5 years' experience. There were no significant differences observed between the other groupings. Comparison of total SABS scores across participant characteristics are presented in Table 4.

**Table 4:** Comparison of mean total SABS scores across participant characteristics.

Variable	Groups	Mean ± SD	Test statistic	*p*‐value
Sex	Male	37.59 ± 6.87	*t*_133_ = 1.56	0.12
	Female	35.08 ± 6.92		
Education level	Undergraduate or equivalent	37.22 ± 7.11	*t*_133_ = 1.93	0.056
	Postgraduate	34.73 ± 6.78		
Sexual health training experience in current role	Yes	32.13 ± 6.98	*t*_133_ = −3.092	0.002*
	No	36.45 ± 6.67		
Sexual health training experience in previous role	Yes	32.73 ± 6.87	*t*_133_ = −2.510	0.013*
	No	36.28 ± 6.80		
Age	18–24	33.33 ± 5.13	*F* = 1.18	0.32
	25–34	38.36 ± 7.75		
	35–44	35.84 ± 6.71		
	45–54	34.43 ± 7.03		
	55–64	34.84 ± 6.83		
	65 and over	37.00 ± 2.65		
Marital status	Married	35.52 ± 6.83	*F* = 0.96	0.43
	Single	37.00 ± 7.82		
	Divorced	35.44 ± 7.06		
	Separated	33.00 ± 3.46		
	Common Law Partnership	32.36 ± 6.17		
Sexual orientation	Straight or heterosexual	35.48 ± 6.91	*F* = 1.069	0.37
	Gay or Lesbian	31.50 ± 8.43		
	Bisexual	35.57 ± 7.79		
	Prefer not to say	41.00 ± 2.65		
Occupation	Nurse	35.27 ± 6.97	*F* = 1.84	0.11
	Nephrologist	35.56 ± 5.92		
	Social worker	31.00		
	Dietician	35.33 ± 7.23		
	Psychologist	19.00		
	Other	38.14 ± 5.40		
Years' experience in renal	Less than 1 year	34.22 ± 4.41	*F* = 3.87	0.01*
	1–5 years	40.00 ± 8.10		
	6–10 years	35.83 ± 5.92		
	More than 10 years	34.49 ± 6.72		

*p < 0.05.

### 3.4 Barriers to Discussing Sexual Health

Mean scores for barriers to discussing sexual health (Table 5) ranged from 2.48 ± 1.23 to 4.76 ± =1.16. The three key barriers to addressing sexual health concerns with patients were a lack of training (4.76 ± 1.16), lack of knowledge (4.28 ± 1.18), and cultural and religious issues (4.23 ± 1.13). In contrast, the three barriers posing the least risk were perceptions that it was someone else's job, (2.48 ± 1.23), holding negative attitudes and beliefs about sexual health (2.82 ± 1.31), and viewing sexual health as a non‐problem for the patient (2.87 ± 1.28).

**Table 5:** Barriers to discussing sexual health in CKD.

Barriers	Mean ± SD	Agree (%)	Disagree (%)
1. Lack of training	4.76 ± 1.16	88.1	11.9
2. Lack of knowledge	4.28 ± 1.18	75.6	24.4
3. Issues related to culture and religion	4.23 ± 1.13	82.3	17.7
4. Not enough time	4.13 ± 1.37	68.2	31.8
5. Older age patient	4.10 ± 1.24	74.8	25.2
6. Personal embarrassment	4.01 ± 1.29	73.4	26.6
7. Patients lack readiness	3.90 ± 1.06	71.8	28.2
8. Fear of offending the patient	3.90 ± 1.24	70.3	29.7
9. Concerns about increasing patients' anxiety and discomfort	3.77 ± 1.20	63.7	36.3
10. Presence of another person that embarrasses the patient to talk	3.55 ± 1.27	56.3	43.7
11. Patient of the opposite sex to you	3.19 ± 1.48	38.6	61.4
12. The too‐large age difference between you and the patient	2.85 ± 1.39	28.1	71.9
13. Sexuality is not seen as a problem for the patient	2.87 ± 1.28	31.9	68.1
14. Negative attitudes and beliefs about sexuality	2.82 ± 1.31	29.6	70.4
15. Someone else's job	2.48 ± 1.23	20.8	79.2

### 3.5 Open‐Text Questions

The open‐text responses offered additional context regarding the challenges of discussing sexual health with adults living with CKD. Some respondents commented that consultants were less likely to address sexual health issues and described the topic as being perceived as taboo. One dietician commented that although discussing sexual health does not fall within their perceived professional role, the topic sometimes arose during conversations with patients.

I am a dietician, it does not seem like I am the best person to start the conversation, but it just seems to come up from time to time in conversation.

Some respondents also highlighted questions regarding where and when discussions about sexual health should take place. Several respondents referred to the importance of privacy, appropriate environments, and the timing of these discussions. Some respondents also expressed uncertainty regarding the optimal timing and frequency for addressing sexual health issues.

I see space and time as a barrier to talk about sex. When is the right time to discuss sex with acute patients and space in regard to where do you hold that conversation, so it preserves the dignity and confidentiality for patient.

Several respondents commented that, although they recognised the importance of addressing patients' sexual health concerns, they lacked confidence in discussing these issues and were uncertain about the information they should provide.

As HCPs, we often believe we have to solve the patients' problems of sexual character but often it just helps the patients to talk with the HCP about their challenges.

Several respondents also highlighted a perceived lack of training and knowledge as a barrier to providing holistic and inclusive sexual healthcare for adults living with CKD.

My great annoyance when looking at the UK renal curriculum is that male sexual health includes ED and Loss of libido. Female ‘sexual health' curriculum only focuses on pregnancy and CKD. These archaic ideas of what female sexual health relates to do not help create new renal consultants who are able to address sexual health issues.

## 4 Discussion

This study provides multidisciplinary perspectives on providing sexual health assessment and support for adults with CKD, including participants from 20 countries. Results of the SABS survey indicate that HCPs in this study possess a positive attitude and belief toward dealing with sexual health issues in adults with CKD. This finding aligns closely to previous research with European nursing students and Australian HCPs involved in brain injury and stroke care, with mean SABS scores of 38.7 and 34.3, respectively (Fraser et al. 2021; Milutinović et al. 2025). In contrast, our findings compare favourably to those of nurses, midwives and students from Lebanon (48.4), Turkey (41.6), Jordan (44.4), Poland (41.7) and China (47.7) (Arikan et al. 2014; Wang et al. 2018; Bdair and Maribbay 2020; Azar et al. 2022; Marcinowicz et al. 2024). Further analysis indicated that mean SABS scores did not significantly differ by age, sex, country, occupation, education level but significant differences were observed based on sexual health training and years of professional experience. The top three barriers identified are lack of training, knowledge, and cultural and religious issues. Sexual health training and the practice of sexual health history taking are rarely provided to renal HCPs. Despite this, most HCPs in this study expressed a desire to receive appropriate sexual health training, supporting prior research with HCPs involved in stroke rehabilitation who highlight the importance of further education and structured training programmes around the topic of sexual health (Low et al. 2022).

Most participants in this study were female, aged between 35 and 54 years. Prior research has reported that older nurses and senior students are more comfortable and possess a more positive attitude to discussing sexual dysfunction issues with patients compared to younger nurses (Senturk Erenel and Cicek Ozdemir 2020; McGrath et al. 2021). Older nurses are also found to handle challenging conversations more effectively due to their broader life experience (Azar et al. 2022; Markowski et al. 2020). In contrast, Marcinowicz et al. (2024) report that younger nurses exhibit greater confidence in their professional knowledge about sexuality. This difference may be attributed to younger nurses holding more liberal views on sex, as opposed to the traditional attitudes held by older nurses (Annerstedt and Glasdam 2019; Thys et al. 2019). However, no significant differences in SABS scores with age were observed in our study. In addition, male HCPs have also been found to possess greater levels of confidence and comfort conducting sexual health assessment with patients compared to their female counterparts (Azar et al. 2022; McGrath et al. 2021). However, the results of our study reported no significant difference between sexes.

The SABS survey revealed that, while HCPs recognised the importance of discussing sexual health with patients and the impact of CKD on sexual function, their ability to address these concerns is hindered by a lack of training and knowledge, leading to limited competence in managing patients' sexual health needs. A lack of competencies among HCPs have been reported, with only 14.2% routinely asking about or resolving sexual health queries for patients with chronic diseases and disabilities (McGrath et al. 2021).

A lack of training was identified as the key barrier to discussing patients' sexual health concerns in CKD across countries and that HCPs have not been offered any training at their workplace, aligning with previous studies (Mckie et al. 2024; Abarca‐Durán et al. 2021; Yodchai et al. 2018; van Ek et al. 2017a). Further, Mckie et al. (2024) reported that most Australian nurses involved in caring for patients with CKD have received no form of sexual health‐related training in their workplace. Notably, this issue is not only reported by nurses involved in CKD care but also by adult patients. Kidney transplant recipients, discussing their experiences on sexual health assessment and management, highlighted that HCPs tended to possess limited sexual health information (Abarca‐Durán et al. 2021). It was recommended by adults who have received a kidney transplant that HCPs should be provided with specific training to discuss sexual health issues more effectively, further supporting the importance of sexual health considerations for this population. Insufficient training and a resulting lack of knowledge regarding sexual health education and assessment among HCPs are commonly reported across a range of chronic conditions. Such conditions include cardiovascular diseases (Inayat et al. 2025; Piegza et al. 2025), oncological diseases (Camejo et al. 2024; Rossi et al. 2025), and within the context of disability (Naghdi‐Dorabati et al. 2024; Letley et al. 2025).

There remains a lack of information on clinical training specifics, such as timing, content, methods, and assessments (McGrath et al. 2021). It is recommended that stakeholders utilise simulated learning and repeated practice in a secure and private setting to enhance skill development (McGrath et al. 2021). Findings from a systematic review on the topic of communication about sexual health in healthcare settings found that HCPs frequently engage in practices such as the use of sensitive terminology, providing generic advice, and using assumptive language (Kelder et al. 2021). Such practices were seen to contribute negatively to the delicacy of discussing sexual health. Rather, HCPs are recommended to adopt language and communication strategies that are more responsive, patient‐centred, and to avoid making assumptions (Kelder et al. 2021). These suggestions may prove beneficial for guiding the development of future training for HCPs to better discuss sensitive topics with patients.

Approximately 82% of participants agreed that culture and religion represent significant barriers when addressing sexual dysfunctions with patients, supporting previous findings (van Ek et al. 2017a, 2017b; Mckie et al. 2024; Wazqar 2020). For instance, in Saudi Arabian communities, discussing sexual health is culturally taboo, making both HCPs and patients uncomfortable. This discomfort leads to fears of offending patients or engaging in inappropriate conversations, making it difficult for HCPs to address sexual health concerns (Wazqar 2020). The influence of culture and religion may help in part to explain the differences observed in SABS scores between the previously mentioned studies with HCPs and nursing students (Arikan et al. 2014; Wang et al. 2018; Bdair and Maribbay 2020; Azar et al. 2022; Fraser et al. 2021; Marcinowicz et al. 2024; Milutinović et al. 2025). Despite these challenges, HCPs can enhance their competence in addressing sensitive topics with patients through education that emphasises cultural differences in healthcare delivery and supplementary training on cultural diversity.

This survey was open to all HCPs; however, participants were mostly from a nursing discipline (84%) and haemodialysis setting (64%). The survey of nurses caring for those with CKD, discussed previously (Mckie et al. 2024), reported that it should be the nurses' responsibility to discuss and address sexual dysfunction with patients. However, patients receive holistic care from multidisciplinary teams, and they may have a conversation with anyone from the care team. Therefore, a multidisciplinary approach is essential due to the limited knowledge and competence reported by the nurses, and that sexual health care in dialysis should not rely solely on individual practitioners. Rather, care in this setting requires a structured multidisciplinary model with clear guidelines and referral systems.

While participants demonstrated relatively more positive attitudes and beliefs towards discussing sexual health, sexual health history‐taking was reported infrequently. This discrepancy suggests that positive attitudes do not necessarily translate into routine clinical practice. The findings indicate that HCPs may recognise the importance of addressing sexual health but remain constrained by practical and organisational barriers, including limited education and training, lack of confidence, time pressures, uncertainty regarding professional responsibility, and concerns about patient discomfort. This attitude‐practice gap has been reported in previous studies (McGrath et al. 2021) and highlights the need for interventions that extend beyond improving attitudes alone to include education, skills development, and organisational support to facilitate the routine integration of sexual health discussions into CKD care.

### 4.1 Strength and Limitations

This global survey included participants from 20 countries and was open to all HCPs involved in providing care to those with CKD, making it the first quantitative study to gather a multidisciplinary renal healthcare team perspective on attitudes, beliefs and barriers to sexual health discussions.

An important limitation of this study is that the achieved sample size (*n* = 135) was substantially lower than the calculated recruitment target (*n* = 385). A further limitation relates to the researcher‐developed survey items; questions were reviewed by the research team to ensure their relevance, clarity, and appropriateness, but formal content validity testing was not undertaken. In addition, the voluntary nature of participant recruitment (e.g. via social media) may have introduced self‐selection bias. HCPs with a greater interest in sexual health or who felt more comfortable discussing sexual health issues may also have been more inclined to participate. Finally, although this study included HCPs from different disciplines and countries, participants were predominantly nurses, HCPs from Denmark and the UK, and those working in haemodialysis settings. This limits the transferability of the findings to other HCP groups, geographical regions, and CKD care settings. Future research involving more diverse and balanced representation is needed.

### 4.2 Implications for Clinical Practice

The findings from this study highlight the need to integrate structured sexual health education into renal training programmes. Such training should focus on improving HCPs' knowledge of sexual health in CKD, developing communication skills and confidence to initiate discussions, and increasing awareness of available support and referral pathways. As the multidisciplinary team plays a key role in the holistic care of adults living with CKD, training should be available to all HCPs involved in renal care. Embedding routine sexual health assessment within holistic CKD care, supported by appropriate education, clinical guidance, and referral pathways, may help normalise conversations, reduce unmet patient needs, and support clinicians to initiate timely and culturally sensitive discussions about sexual health. In turn, this can enhance the quality of care delivered to adults living with CKD.

## 5 Conclusion

HCPs' attitudes and beliefs towards discussing sexual health with patients is positive, but further training to improve competencies is desired. Further research is required to gain a comprehensive understanding of the perspectives of various HCPs in renal care, not just nurses. The primary barrier requiring urgent attention is the lack of training, highlighting the need for stakeholder engagement to develop appropriate educational strategies related to sexual health.

## Author Contributions

**Clare McKeaveney:** Principal Project Leader, conceived study, participated in design and coordination, read and approved the final manuscript. **Xingge Sun:** Analysed the data, helped to draft manuscript and approved the final manuscript. **James McMahon:** Analysed the data, helped to draft manuscript and approved the final manuscript. **Ann Bonner:** helped to draft manuscript and approved the final manuscript. **Maoliosa Donald:** helped to draft manuscript and approved the final manuscript. **Sandra Dumanski:** helped to draft manuscript and approved the final manuscript. **Meghan Elliott:** helped to draft manuscript and approved the final manuscript. **Jeanette Finderup:** helped to draft the manuscript and approved the final manuscript.

## Funding

The authors have nothing to report.

## Disclosure

For the purpose of open access, the authors have applied for a Creative Commons Attribution (CC BY) license to any Author Accepted Manuscript arising from this submission.

## Ethics Statement

Ethical approval was received from the Faculty of Medicine, Health, and Life Sciences. Research Ethics Committee, Queen's University, Belfast (MHLS 22_139).

## Conflicts of Interest

The authors declare no conflicts of interest.

## Data Availability

The data that support the findings of this study are available on request from the corresponding author. The data are not publicly available due to privacy or ethical restrictions.
